# Semiconducting hematite facilitates microbial and abiotic reduction of chromium

**DOI:** 10.1038/s41598-022-12824-y

**Published:** 2022-05-31

**Authors:** Michael A. Chen, Neha Mehta, Benjamin D. Kocar

**Affiliations:** 1 Parsons Laboratory, Department of Civil and Environmental Engineering, MIT, 15 Vassar St., Cambridge, MA 02139 USA; 2 Department of Earth and Environmental Sciences, University of Minnesota, 116 Church St. SE, Minneapolis, MN 55455 USA; 3 Institut de Minéralogie, de Physique des Matériaux, et de Cosmochimie, Sorbonne Universités, 75005 Paris, France; 4 Environmental Laboratory, US Army Engineer Research & Development Center, 3909 Halls Ferry Road, Vicksburg, MS 39180 USA

**Keywords:** Biogeochemistry, Chemistry, Environmental chemistry

## Abstract

Semi-conducting Fe oxide minerals, such as hematite, are well known to influence the fate of contaminants and nutrients in many environmental settings through sorption and release of Fe(II) resulting from microbial or abiotic reduction. Studies of Fe oxide reduction by adsorbed Fe(II) have demonstrated that reduction of Fe(III) at one mineral surface can result in the release of Fe(II) on a different one. This process is termed “Fe(II) catalyzed recrystallization” and is believed to be the result of electron transfer through semi-conducting Fe (hydr)oxides. While it is well understood that Fe(II) plays a central role in redox cycling of elements, the environmental implications of Fe(II) catalyzed recrystallization require further exploration. Here, we demonstrate that hematite links physically separated redox reactions by conducting the electrons involved in those reactions. This is shown using an electrochemical setup where Cr reduction is coupled with a potentiostat or *Shewanella putrefaciens*, a metal reducing microbe, where electrons donated to hematite produce Fe(II) that ultimately reduces Cr. This work demonstrates that mineral semi-conductivity may provide an additional avenue for redox chemistry to occur in natural soils and sediments, because these minerals can link redox active reactants that could not otherwise react due to physical separation.

## Introduction

Metal (hydr)oxides are important substrates that constrain contaminant and nutrient fate in soil-sedimentary systems through sorption and redox reactions^1–11^. Hence, dissolution and transformation of these metal hydr(oxides) is an integral component of soil geochemistry, and necessary to understand major nutrient and contaminant cycling. For example, iron oxides may play a central role in the nutrient cycling for deep biosphere habitats, such as mines, where limited carbon and oxygen but abundant Fe favor the growth of metal reducing bacteria^12^. These solids are also well recognized sorbents for a variety of contaminants, including arsenic, radium, and chromium^10, 11, 13, 14^.Hematite (Fe_2_O_3_) has been thoroughly studied due to its environmental ubiquity, extensive sorption of many solutes of interest, and sensitivity to redox conditions. Reductive dissolution of hematite leads to the release of Fe(II), which can then react with organic matter or other metals in solution to drive further redox reactions^15–19^.

Hematite reduction may occur through transport and sorption of reduced soluble constituents such as sulfide or quinones, which can be abiotic or biotic in origin, or by direct conduction of electrons from a dissimilatory metal reducing bacteria (DMRB) at the surface^20–22^. Reduction by DMRB is one of the most important controls on Fe redox cycling because DMRB rely on hematite and other Fe (hydr)oxides as a primary electron sink for their anoxic metabolism. A variety of mechanisms allow microbes to access these solid electron sinks, which include the use of microbial nanowires, release of electron carriers, such as cytochrome *c*, as well as direct contact with minerals by surface colonization^21, 23–26^. Metal reducing bacteria use these mechanisms to access electron acceptors at ranges as large as 10 µm to 20 µm, as well as access nanopores that are too small for cells to enter^25,27^. DMRB have also been observed to use these mechanisms with conductive electrodes held at a reducing potential, which has provided the basis for development of microbial fuel cells^28, 29^. While terminal electron accepting processes between DMRB and metal hydroxides are important from a metabolic perspective, they also facilitate mineralogical alterations that influence the cycling of metals/nutrients.

Studies using isotopically labeled Fe(II) have demonstrated that the semi-conductivity of hematite (and other similar Fe oxides) results in a cycling of Fe between the aqueous and solid phase through atom exchange processes and mineral recrystallization^30–32^. Investigation of this phenomena revealed that following the sorption of Fe^57^ enriched aqueous Fe(II) to one surface of the mineral, Fe^56^(II) would be released on another surface, resulting in isotopic equilibration of the solution with the mineral without alteration of the mineral structure or crystallinity^33, 34^. This phenomena, termed Fe(II) catalyzed recrystallization, is postulated to arise from conduction of the electron donated by sorbed Fe(II) to another point on the mineral, which is facilitated by the mineral’s semi-conductivity^32^. This process is well understood to impact the environmental cycling of Fe, and may also impact the cycling of other metals and nutrients^35^. For example, Fe(II) catalyzed recrystallization of Fe solids in the presence of U boosted incorporation of U into the Fe solid over a 90 day period, while Fe(II) catalyzed recrystallization of Mn, Ni, or Zn doped Fe solids resulted in enhanced release of those trace metals over 5 to 10 days^36, 37^. The Fe(II) produced by DMRB during bacterial metal reduction has also been shown to enable Fe(II) catalyzed recrystallization of Fe solids, further adding to the web of interactions between these solids and DMRB^38^. Furthermore, other redox active species produced by microbial respiration, such as nitrogen or sulfur species, may also readily drive this cycling of hematite^20, 39, 40^. These studies have collectively demonstrated that naturally occurring redox reactions will strongly influence the composition of Fe solids beyond the surface.

While electron conduction, and its impact on the incorporation and release of elements, has been explored in detail for semi-conducting Fe (hydr)oxides such as hematite or goethite, it remains unclear whether electron transfer through Fe oxides may affect other redox reactions that occur on the surface or near an Fe oxide. Specifically, the current understanding of this process suggests that an electron donated to a semi-conductive Fe (hydr)oxide mineral surface (such as during bacterial metal reduction) could be conducted to another location where a subsequent redox reaction could occur. If so, the Fe (hydr)oxide would electrically link the two reactions, thus coupling the two reactions. This possibility was initially suggested in initial studies of Fe(II) catalyzed recrystallization, where dissolution and precipitation of hematite were observed on physically distinct crystallographic planes indicating the conduction of electrons from one surface to another^32^. A recent study has also explored this possibility with the model compound, cytochrome *c*, which was used to represent biotic Fe(II) oxidation, and found that electrons from Fe(II) sorbed to hematite would reduce cytochrome *c* by conduction through the hematite^41^. However, further investigation is required to understand if Fe (hydr)oxide conductivity could lead to the coupling of redox reactions via Fe oxide conductivity.

To determine whether electron conduction through iron (hydr)oxides may link redox reactions, we investigate whether a DMRB or other electron source would be able to affect reduction of an electron acceptor, here, Cr, via conduction through hematite. Chromium is chosen as the terminal electron acceptor for these systems owing to its relevance for contaminated soils and sediments as well as its capacity to disambiguate direct microbial reduction from indirect reduction by Fe(II), thus serving as a chemical probe for the fate of electrons conducted by hematite. Cr(VI) is a carcinogenic and highly soluble metal, and is often produced and released to soils and groundwater through industrial activities, while Cr(III) is relatively insoluble and has minimal toxicity^42–46^. Hexavalent Cr can be directly reduced by metal reducing microbes to form Cr(OH)_3_, but can alternatively be reduced by Fe(II) to form mixed Fe/Cr hydroxide solids, which are typically in the form Cr_1–x_Fe_x_(OH)_3_, where x is as large as 0.75^1, 44, 46, 47^. The dependence of Cr solid composition on reductant has previously been leveraged to understand changes in Fe(II) activity during the redox cycling of goethite, and is used here to a similar effect^48^. Numerous studies have also shown redox coupling between pairs of these components (hematite, Cr, DMRB), however, it remains unclear how reduction would proceed if all three are present simultaneously. The overarching goal of this work, therefore, was to determine how hematite conductivity can link spatially segregated redox reactions, where the reactions are reduction of Fe by DMRB or abiotically, and reduction of Cr.

## Results

**Figure 1:**
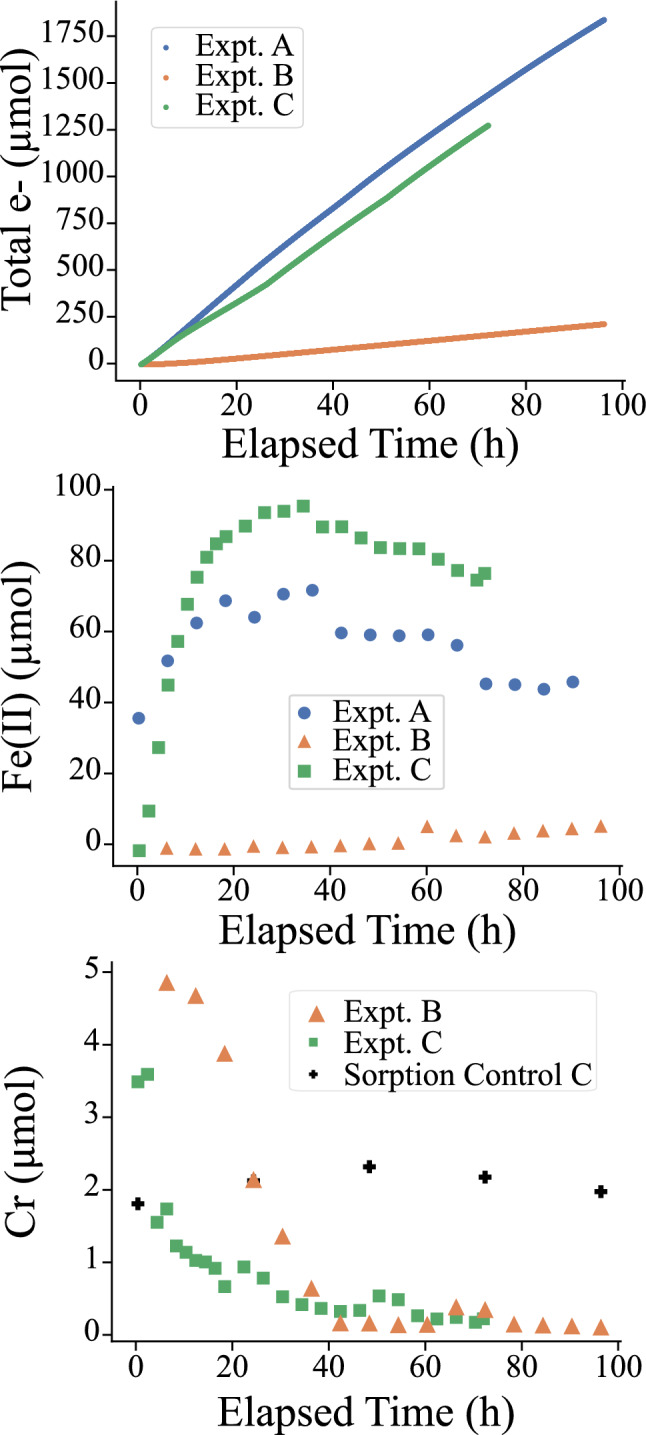
Graphs showing trends of electron transfer, dissolved Fe(II), and dissolved Cr in potentiostatic experiments. In all experiments, the hematite electrode is poised to − 1000 mV vs. Ag/AgCl.

Experiments to test for how hematite can mediate reduction of Cr by an abiotic or biotic electron source were performed using a two chamber electrochemical cell with a cation exchange membrane, a natural specular hematite electrode, and carbon electrode. Cr reduction can only occur if electrons are conducted through the hematite electrode because the cation exchange membrane prevents physical migration of chromium to the electron source. Table 1 summarizes the different electron sources and hematite electrode shapes used, as well as the average rate of Cr removal, average current, and Fe(II) production for the experiments. These rates are simply the amount of change in the amount of Cr, e^−^, of Fe(II) over a predefined time period. In experiments containing Cr (B, C, D), the time period is from the initiation of the experiment until Cr concentrations are stable, and is indicated in Table 1. In experiment A, without Cr, the window is from the start of experiment to the time where maximum Fe(II) is observed (35.9 h). Control experiments (Fig. 1 and SI Figure S3) without reduction (i.e. without a potentiostat or *S. putrefaciens*) showed that the impact of sorption on Cr removal was minimal and that electron transfer would not occur without the inclusion of the potentiostat or the metal reducing bacteria (SI for details).

**Table 1:** Summary of experimental configurations. Uncertainties are the largest of either the ICP-MS measurement error derived from the standard error of the calibration curve or standard deviation of the replicate samples taken from the experiment.

Experiment ID	Electron source	Hematite electrode	Initial Cr concentration (µM)	Cr removal Rate (µmol/s)	Average Current (µA)	Fe(II) production rate (µmol/s)	Elapsed time for rate calculation (hrs)
A	Potentiostat	Bulk	N.D	N/A	5.9E-3	2.8E-4	36
B	Potentiostat	Bulk	14.3 ± 0.8	3.6E-5	6.1E-4	7.7E-7	36
C	Potentiostat	Thin section	11.8 ± 2.0	2.5E-5	4.8E-3	7.8E-4	34
D	*S. putrefaciens*	Bulk	22.5 ± 1.1	4.9E-6	9.3E-6	N/A	313

The average rate of Cr production, average current, and production of Fe(II) are also given here. The time period considered in experiment A is from the experiment start to the point where the maximum Fe(II) is observed. The elapsed experimental time used to calculate these rates is also stated for comparison.

### Dissolved Cr and Fe Time Series

 The total amount of Fe, Cr, and electron transfer was measured during potentiostatic experiments (A, B and C from Table 1) and is shown in Fig. 1 for the chamber containing the hematite electrode. The Fe observed in solution was exclusively Fe(II) except in experiment A; the distribution of Fe(II) and Fe(III) in solution for that experiment is discussed further in the SI. Measurements for the chamber containing the carbon counter electrode can also be found in the SI. Table 1 also presents the average rate of Cr removal, Fe(II) production, and current. Expt. A, which used no Cr, and Expt. C, which used Cr and a thin section hematite electrode, showed orders of magnitude larger Fe(II) release rates, as well as significantly larger current. In potentiostatic experiments with Cr, dissolved amounts of Cr decreased by 96% (exp B) and 89% (exp C), corresponding to similar rates of removal. After that time, the dissolved Cr amount remained stable but not below detection levels (1 ug/L corresponding to 6 nmol Cr in a typical experiment). No Cr was detected in the carbon electrode chamber at any time point during the experiment. In experiment B, near complete Cr removal is observed prior to any appearance of Fe(II), while in experiment C, Cr removal and Fe(II) release occur simultaneously.

**Figure 2:**
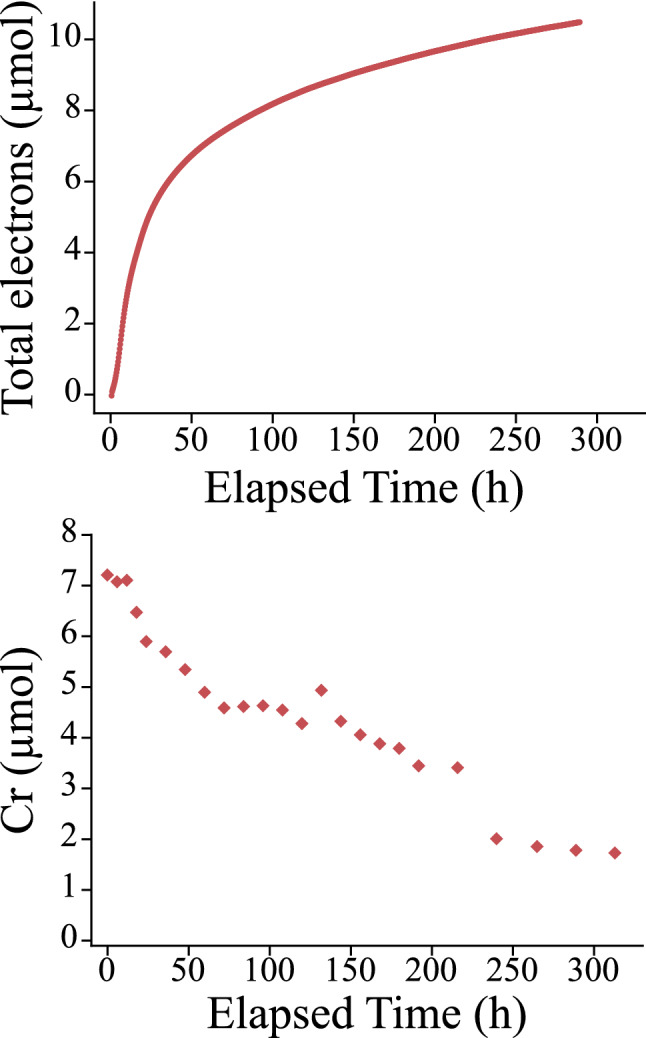
Biotic experimental results. Plots of current transfer and Cr changes in the hematite chamber from experiment D with *S. putrefaciens* as the current generator.

The results of the biotic experiment (D), where *S. putrefaciens* provides electrons for reduction are shown in Fig. 2. The time series of current, *S. putrefaciens* OD measurements, and corresponding measurements of solutes in the carbon electrode chamber are presented in the SI. Fe was below the detection limit of the ferrozine method in these experiments, which was 0.015 mg/L, corresponding to 0.08 µmol of Fe in a typical experiment. In contrast with potentiostatic experiments, not all Cr was removed, and the rate of Cr removal in this experiment was markedly slower than that observed in potentiostatic experiments B and C.

### Surface chemistry results

**Figure 3:**
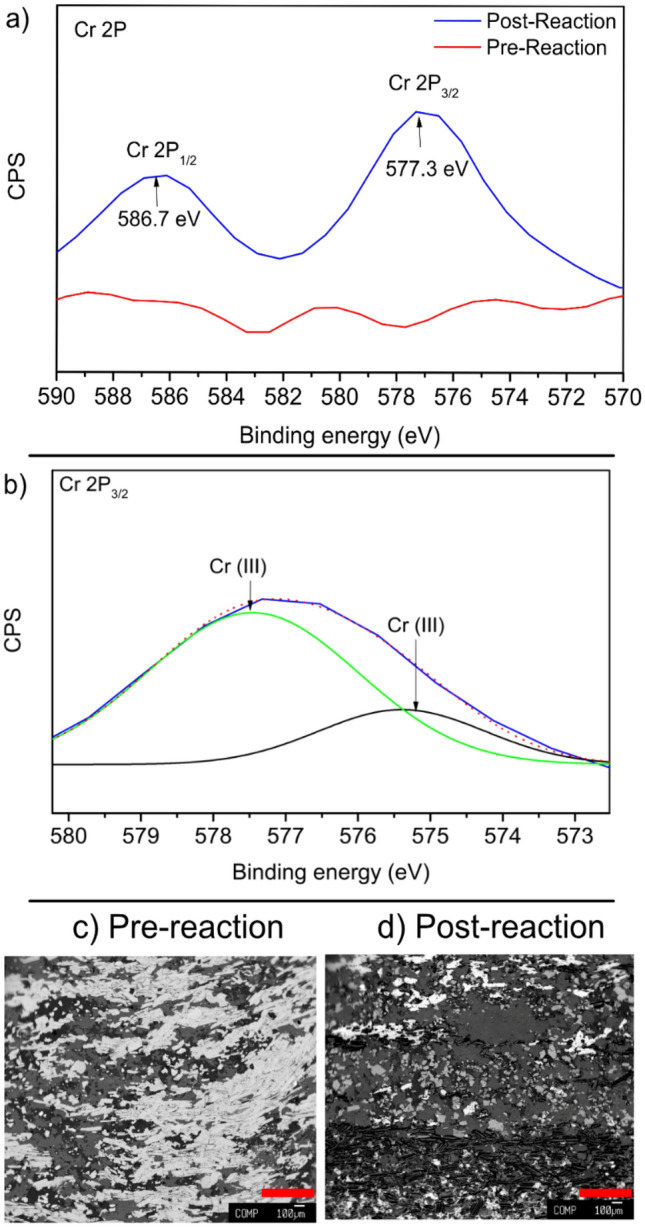
Results of hematite thin electrode surface analysis (**a**) XPS results for a representative point near the Cr for both unreacted and reacted electrodes, which show the appearance of Cr on the surface of the reacted electrode. (**b**) Fitting results for the Cr 2P3/2 peak, which indicate the presence of exclusively Cr(III) compounds. (**c**) Electron backscatter image of a hematite thin section electrode prior to reaction. (**d**) Electron backscatter image of a hematite thin section electrode after reaction, showing transformation of the surface.

 XPS spectra and electron backscatter images were collected to characterize surface changes of a thin electrode after use in the potentiostatic experiment C. Figure 3 shows Cr region XPS spectra for representative points on the surface of the unreacted sample when compared to the one used in experiment C. The main Cr 2p_3/2_ peaks were located at a binding energy of ∼577 eV, which corresponds to Cr(III), based on values ranging between 577.0 and 578.0 eV for Cr 2p_3/2_ reported for Cr(III)-containing materials^49, 50^. The Cr2p_1/2_ signal located at 586.7 eV also supports the existence of Cr(III). Cr(VI) species like CrO_3_ characterized by higher binding energies; 580.0–580.5 eV and 589.0–590.0 eV, since the hexavalent form draws electrons more strongly than the trivalent form. These were clearly absent in the XPS spectra of electrode acquired here. Full XPS spectra measured at each location on the electrode before and after use are presented in the supplementary information. Figure 3 also shows that the surface of the hematite observed in the electron backscatter images was highly heterogeneous, consisting of multiple solids, some of which are aluminosilicate solids as determined by collected EDS spectra (SI). In comparing the representative locations from the reacted and unreacted samples, the major difference is the disappearance of the white mineral, which primarily contained Fe according to the EDS spectra. EDS spectra did not show explicit Cr solids, however, based on the µmol amounts of Cr used in the experiment, this is likely due to insufficient Cr to create a signal. To quantify the potential impact of the observed surficial heterogeneity on hematite electron transfer, the hematite resistivity was measured in three samples similar to those used as bulk electrodes (i.e. in experiments A, B, and D), to characterize how this observed heterogeneity would impact electron transfer (SI for further details). These measurements found the resistivity of the hematite used in this study varied widely, ranging from 500 Ωm to nearly 11,000 Ωm. The observation of these varied solids is in alignment with these measurements of the hematite resistivity, as the bulk resistivity of a specific sample will represent an aggregation of the individual solids comprising that sample.

## Discussion

In both the biotic experiments and abiotic experiments, Cr was removed from solution, though at differing rates. Since the control experiments clearly demonstrated that sorption was not affecting the Cr concentrations and is supported by the lack of Cr(VI) observed in XPS spectra for experiment C, the only driver for this observed behavior must be reductive precipitation, which must be associated with the current delivered. This follows from the normal speciation of Cr(VI) and Cr(III) at pH 7, where Cr(VI) will remain in solution while Cr(III) will be rapidly removed from solution by precipitation^1, 44, 47^. This is corroborated by the emergence of Fe(II) in potentiostatic experiments, which is a result of hematite reduction by the delivered current, and variations in that current yield variations in the observed Fe(II) released. The variations in turn are the result of the varied resistivity of the natural source hematite instead of a higher purity synthesized hematite. The difference in current magnitude between the biotic and potentiostatic experiments also suggest that the amount of current is linked with Cr reduction, though the linkage is less clear. In particular, the current differences are not able to discriminate between direct Cr reduction by electrons conducted through the hematite and indirect reduction by the produced Fe(II). Both cases will link total electrons delivered to the amount of Cr reduction observed, but these different mechanisms have markedly different implications for the mechanisms at play in these experiments.

Cr, in addition to being a contaminant of concern in many groundwater systems, also then serves as a chemical probe for discriminating between direct reduction and indirect reduction by Fe(II), particularly when considering the ratios of Cr/Fe observed by XPS. This is readily calculated from the XPS data by comparing the ratios of Cr to Fe observed on the surface of the thin section hematite electrode used in experiment C (Fig. 3). The ratio varies throughout the sample, with Cr/Fe ratios ranging from 0.0 to 0.5 (SI figure S2). Previous studies of Cr reduction in the presence of Fe showed that Cr will form a mixed Cr/Fe solid when exposed to Fe(II), with Cr/Fe ratios as low as 0.33, as opposed to forming a pure phase Cr(OH)_3_ when directly reduced, which here would result in a very large Cr/Fe ratio^46–48^. The general formulae for these minerals is Cr_1−*x*_Fe_*x*_(OH)_3_ · *n*H_2_O, corresponding to mixed Cr/Fe hydroxides. Multiple locations on the reacted hematite electrode (5 of 21) have Fe/Cr ratios within the range of these types of mixed Cr/Fe solid. The largest Cr/Fe ratio observed in the XPS results is 0.5, which would most likely represent these mixed Cr/Fe solid previously observed, rather than a pure Cr phase, thus suggesting that Cr was reduced by Fe(II) at all locations, rather than reduction by electrons conducted to surface associated Cr(VI). This solid would correspond to a stoichiometry of Cr_0.33_Fe_0.66_(OH)_3_ · *n*H_2_O. Similarly, the peak fitting results indicate that all Cr on the surface is Cr(III), further reinforcing that co-precipitation with Fe has occurred, rather than sorption of Cr(VI). XPS studies of Cr reduction by Fe(II) have previously shown that it is difficult to discriminate between pure Cr(OH)_3_ solids and mixed Fe/Cr minerals by shifts in the Cr 2p spectra alone, and generally found that during reduction of Cr by Fe(II), mixed Fe/Cr solids predominantly form^51^. These results are consistent with other studies as well, where Cr was observed to form mixed Cr/Fe solids following Cr(VI) reduction by Fe(II) in solution^46, 47^. The indirect reduction process also explains why the timing of Fe(II) release and Cr reduction vary between experiments (i.e. that in expt. B Cr reduction precedes Fe(II) release, while in expt. C, they occur simultaneously). If the delivered current produces Fe(II) at a rate faster than Cr reduction by Fe(II) can occur, then both Cr removal and Fe(II) release would occur simultaneously. The variations in delivered current are also in alignment with this observed behavior. Lastly, indirect Fe reduction of Cr would explain why no dissolved Fe(II) is observed in the biotic experiment D, as the small amount of Fe(II) produced by the lower current has entirely driven reduction of Cr.

To support a mechanistic conceptual model, Cr removal rates can be compared against the current to the hematite electrode. Since Cr reduction is the only pathway that results in removal from solution, the quantity of Cr removed is considering using the half reaction:

1 $${\text{Cr}}\left( {{\text{VI}}} \right)\left( {{\text{aq}}} \right) + 3{\text{e}}{ - } \leftrightarrow {\text{Cr}}\left( {{\text{III}}} \right)\left( {\text{s}} \right)$$

Accordingly, each mol of Cr removed corresponds to 3 mol of electrons transferred to the hematite electrode. Multiplying the Cr removal rate given in Table 1 by 3 and comparing against the current should then give the fraction of current which contributes to Cr reduction. In potentiostatic experiments B, Cr reduction only accounts for 17.7% of the delivered current, while in experiment C it accounts for only 1.6% of the delivered current. The remaining current likely then results in Fe reduction, which is discussed below. In contrast with the abiotic experiments, the amount of Cr reduction observed in biotic experiments exceeds the delivered electrons, with the amount of Cr reduction being 157.1% of the delivered current. Based on the collected data, the reasons for such anomalously high Cr reduction in the biotic experiment remains unexplained. One possibility is that Fe/Cr solids that have formed due to reduction are able to uptake additional Cr through sorption. Sorption of Cr to Fe and mixed Fe/Cr solids has been observed in cyclic studies of Cr reduction by microbes in the presence of Fe^47, 52^. While in those studies, DMRB were able to completely reduce all available Cr, some amount of surficial sorption was observed, and could readily explain the Cr discrepancies, since the discrepancy corresponds to only a few µmol of Cr. Similarly, the reduction of Fe will alter the surface of the hematite electrode, as evidenced by the electron backscatter images in Fig. 3, which may further enhance sorption processes either by enhancing the surface area relative to the original polished surface, or by the formation of solids with higher sorption affinity for Cr. The reason this was likely not observed in potentiostatic experiment B and C is due to the large amount of current that was delivered to the electrode, which would lead to complete Cr reduction regardless of if it had time to sorb to the altered surface or not.

A similar process can be applied to Fe to complete the balance of electrons transferred, in which each mol of Fe(II) measured accounts for a mol of the transferred electrons, following the half reaction:

2 $${\text{Fe}}\left( {{\text{III}}} \right)\,\left( {\text{s}} \right) + {\text{e}}{ - } \leftrightarrow {\text{Fe}}\left( {{\text{II}}} \right)$$

For the biotic experiment D, no dissolved Fe is measured, and all current is accounted for. However, as evident in Table 1, the Fe(II) production rate in potentiostatic experiments is significantly smaller than the total current. The only other potential redox reaction in this system would be splitting of water into hydrogen and oxygen, however, no gas evolution was observed in any experiment. Any measured current beyond that which contributes to Cr reduction must, therefore, result in Fe reduction. The electron backscatter images support this possibility, as the dramatic change in the surface between the reacted and unreacted electrodes could readily occur due to reductive dissolution of Fe. This does not, however, constrain the fate of all Fe(II) that would have been produced by the provided current.

The fate of Fe(II) cannot be completely determined directly from the analyses performed here, but there are a few reasonable possibilities: First, Fe(II) generated remains sorbed to the hematite electrode surface. Fe(II) sorption to Fe (oxy)hydroxides is well documented and has previously been observed during cyclic oxidation and reduction of goethite^30^. In those experiments, reduced Fe(II) persisted even throughout oxic conditions by associating with the surface, so it is very likely at least some amount of Fe(II) would be retained on the surface^48,53^. Second, the dissolved Fe(II) may form a precipitate on the hematite surface. The formation of these types of solids has been observed in other studies of hematite redox cycling, and is reasonable to expect here^9, 54, 55^. Both sorption of reduced Fe as well as precipitation of other Fe minerals is reasonable here and would readily close the balance of electrons, however, further studies are needed that explicitly track Fe fate, such as through the use of an isotopically labeled Fe tracer. PIPES, the buffer used in these experiments, is well understood to enhance solution complexation of Fe(II) and would need to also be carefully considered in Fe tracking experiments^56^. Despite this limitation, the fate of Cr, Fe, and the current in this system is sufficiently constrained to propose a mechanism that explains the trends and behavior observed in these experiments.

**Figure 4:**
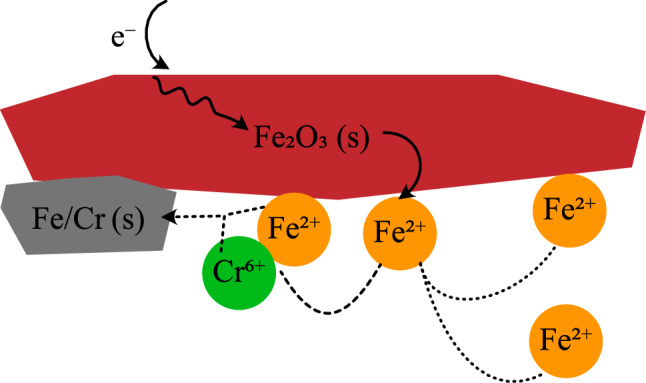
Schematic of conceptual model which illustrates how electrons interact with hematite to reduce Fe and Cr. Conducted electrons from an arbitrary source produce Fe(II) which can either reduce Cr to form a mixed Fe/Cr solid or remain as Fe(II) to distribute between sorbed and dissolved species.

Figure 4 gives a proposed conceptual model that describes the fate of Cr, Fe, and electrons in this experimental system. Electrons that arrive at the hematite electrode, regardless of their origin, induce reductive dissolution of hematite, thereby producing Fe(II) at the interface of the hematite electrode and solution. The resulting Fe(II) reduces Cr to form mixed Fe/Cr solids, with perhaps some direct reduction of Cr(VI) to Cr(III). When the rate of Cr reduction is slower than the delivered current or all Cr has been reductively precipitated, excess Fe(II) is produced. This Fe can be found both in solution and associated with the hematite electrode. This proposed mechanism is a natural extension of the established mechanism behind Fe(II) catalyzed recrystallization; the main difference here from those works is that there is a separate source of electrons as well as a terminal electron acceptor instead of Fe(II) as electron source and terminal electron acceptor^31–33, 41^. These results demonstrate that hematite, or other semi-conductive Fe minerals, may serve a broader role in controlling redox chemistry in natural soils.

DMRB are well established to rely on Fe hydr(oxides), such as hematite, as an electron acceptor during anaerobic metabolism, and the results here expand the potential interactions between an Fe oxide and DMRB. The biotic experimental results clearly demonstrated that hematite created a coupling between microbial metabolism of lactate and Cr reduction, implying that semiconductive phases, such as hematite, may enable coupling of other redox processes. One prominent example where this will be relevant is direct interspecies electron transfer (DIET), where one bacterial species will donate the electrons from their metabolism to another species^57, 58^ . The observed coupling of reactions here supports the possibility that bacterial communities could use hematite as a mediator for these electron transfers. Frequently, hematite reduction is preceded by microbial reduction of sulfate or nitrate; the results here are not specific to microbial iron reduction, and semi-conductive hematite may also either mediate this microbial reduction, or facilitate redox reactions of the resulting nitrogen and sulfur species^39, 40, 59^. These results are also in good alignment with assertions that bacteria can use conductive substrates (i.e. minerals, other bacteria) to form bacterially active networks, as metal reducing bacteria here used hematite to access Cr as a terminal electron acceptor^21, 58, 60–62^. Further investigation is required to understand the prevalence of these networks in natural soils and their importance to bacterial growth in natural settings.

Some of the earliest electrochemical studies, where sacrificial anodes were used to prevent oxidation of metal ship components, established that electrical contact was sufficient to couple redox reactions of different metals that were otherwise separated^63, 64^. This work follows in the footsteps of that foundational work: hematite here has enabled spatially segregated redox reactions between a metal reducing bacteria or other electron source, and Cr. The main components of this system, a semiconductive Fe oxide, an Fe reducing reaction, and an Fe oxidizing reaction, are ubiquitous in the environment, suggesting that the prevalence of this kind of linkage is more prevalent than has been previously reported. The results of this work, however, outline only the first steps of broader understanding of how mineral conductivity may influence groundwater chemistry. Fe(II) forms favorable redox couples with other priority contaminants such as U or As, and this mechanism could play a role in the remediation or natural cycling of those contaminants^3, 15, 65–67^. Fe(II) catalyzed recrystallization has been demonstrated for goethite, another semi-conducting Fe mineral, thus the results here also imply that this conduction mechanism may be more broadly applicable wherever Fe cycling occurs. Given the ubiquity of semi-conductive Fe (oxide) solids in natural soils, it is likely that this type of coupling is likely widespread wherever redox gradients intersect these solid materials, though its importance may be affected by mineral or other organic coatings that ultimately constrain the conductivity of these minerals. One locale where this mechanism may be prevalent is in deep mines, where abundant semiconductive ores may allow native microbial communities to access distant electron acceptors and nutrients^12, 68^. Further investigation is needed, however, to demonstrate the range of biogeochemical systems where this mechanism is relevant, further understand the influence of these processes on hematite surface chemistry and illustrate the importance of this mechanism in natural soils and groundwater.

## Methods

**Figure 5:**
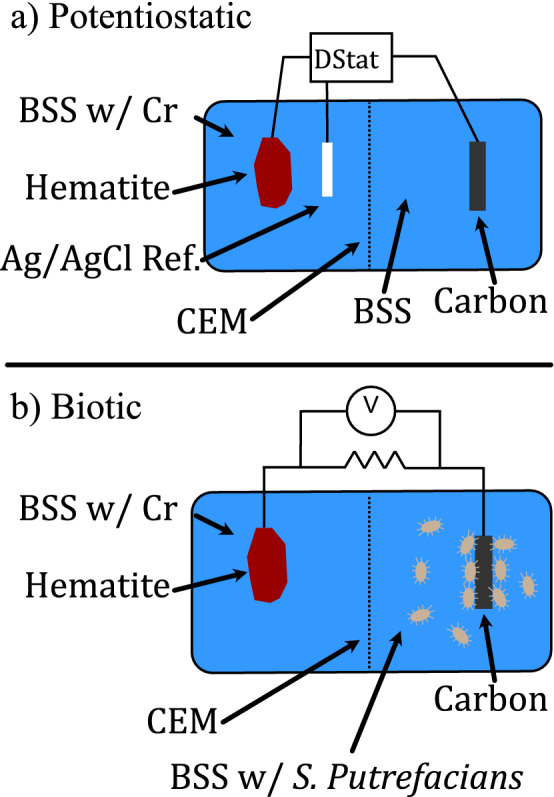
Experimental schematics of electrochemical setup with a cation exchange membrane (CEM). (**a**) Schematic of potentiostatic experiments. (**b**) Schematic of biotic experiment using *S. putrefaciens.*

Two electrochemical experimental systems were used to examine how hematite mediates the coupling between Cr reduction and bacterial metabolism: in the first, a potentiostat was used to poise the voltage of an experimental hematite electrode, and in the second, the DMRB *S. putrefaciens* poises the electrode potential based on available carbon source, here, lactate, and electron acceptors, Fe and/or Cr (Fig. 5). The potentiostat (open-source, DStat) was built following the given^69^ instructions. Both experiments used a 3D printed two chamber system, a polished specular hematite electrode (sample from Republic, MI, Ward’s Scientific,), graphite electrode, and a coated paper cation exchange membrane (CMI-7000S, Membranes International, Inc.) which was used to separate the electrodes while allowing for ion migration necessary to maintain charge balance during electron transfer. This experimental set up was chosen because it allowed for physical separation of bacteria and Cr, thus hematite would serve as the mediator for all redox reactions that occur. The potentiostatic experiments additionally used a Ag/AgCl reference electrode to accurately poise voltages. All electrodes were fabricated in house except for one type of hematite electrode: a thin section of specular hematite mounted on silver epoxy, which was prepared by Spectrum Petrographics, Inc. Further details on the fabrication and handling of the electrodes and the chamber are included in the supplemental information.

All experimental solutions used in these experiments were made in 18MΩ water with ACS grade chemicals. To ensure that *S. putrefaciens* would perform metal reduction (as opposed to respiring oxygen) and to prevent premature oxidation of Fe or Cr, all experiments were performed in an anoxic glove bag (CoyLabs, 2–3% H_2_, balance N_2_). Solutions were prepared in normal laboratory air, purged with N_2_ for at least 1 h, and then immediately transferred to the anoxic glove bag. Measured oxygen levels in the glove bag were always below 1 ppm throughout experiments. A background basal salts solution (BSS) with a sodium PIPES buffer (50 mM) was used to fill the chambers for all experiments^3^. A buffer was included in the BSS because hematite dissolution is known to increase solution pH. Initial experimental pHs were 7*.*1 ± 0*.*2, and the only significant pH increase (0.8 pH units) was observed in an experiment where no Cr was included, likely due to hematite dissolution (Table 1, expt. A). Cr was added to experiments through a small volume spike potassium chromate stock (50 mM) to the chamber containing the hematite electrode, and there was no evidence of Cr migration across the cation exchange membrane in any experiment.

The primary differences between the two experiments were their electrical configuration (Fig. 5, Table 1). For potentiostatic experiments, the potentiostat was connected to the hematite, graphite, and reference electrode and was controlled using a Python script to poise the hematite working electrode at − 1000 mV vs. the Ag/AgCl reference electrode^69, 70^. In these experiments, the graphite electrode served as a counter electrode. Both the thin section hematite electrode and “bulk” hematite electrodes were used in these experiments as working electrodes. When using a thin section electrode, care was taken to ensure that the only conductive surface in contact with the experimental solution was hematite by sealing the electrode edges and maintaining the point of electrical contact in air. In potentiostatic experiments, currents provided to the working electrode were directly reported by the potentiostat using a built in measurement circuit^69^. For biotic experiments, the graphite and hematite electrodes were simply connected with a 100 Ω resistor, which provided sufficient resistance to allow for current measurement while not overly inhibiting electron transfer from *S. putrefaciens*. For biotic experiments, a Keithley 2100 multimeter controlled by a Python script was used to measure and log voltages measured across the connected resistor, which was then converted to current^70^. Total amounts of electron transfer were calculated by integrating measured current in time using a trapezoidal method, as implemented in the NumPy package^71^.

Sampling of experimental systems involved collection of aqueous samples, and in the potentiostatic experiment with a thin electrode, collection of the electrode itself. Aqueous samples collected from biotic experiments were filtered on 0.22 um PES syringe filters to remove any possible bacteria. All samples were acidified within 12 h after collection by the addition of 3 drops of HCl (6 N), and were stored in plastic falcon tubes. Total solution Cr concentrations were measured in these samples using a Perkin Elmer NexIon 300-D ICP-MS, while dissolved Fe(II) and Fe(III) in these samples was measured through the Ferrozine method. Concentrations of dissolved constituents were then converted to mole amounts by using the solution volume calculated by changes in experimental mass^72, 73^. Further analytical details are available in the supplementary information.

*S. putrefaciens*, strain CN-32 (ATCC), was grown up from 50% glycerol stocks frozen at − 80 °C, in tryptic soy broth over a 16 h period and harvested in the exponential growth phase by centrifugation and rinsing with BSS 3 times. On the final rinse, anoxic BSS was used. All solutions used in biotic experiments were autoclaved at 121 °C prior to use, and all experimental surfaces (electrodes, chamber interiors, cation exchange membrane, etc.) were sterilized by either rinsing with 70% ethanol and drying in a biosafety cabinet, or by exposure to UV light for a minimum of 60 s. Bacteria were provided 10 mM lactate as a carbon source, and initial cell concentrations in experiments were 10^7^–10^8^ cells*/*mL, measured using the optical density at 660 nm as calibrated to measurements with a Guava flow cytometer.

The surface of the reacted and unreacted hematite electrodes was examined and compared with unreacted electrode surfaces. Once the reacted electrode had been used in an experiment, it was dried and kept in an anoxic environment until just before analysis, while the unreacted electrode was used as is after polishing. An EOL-JXA-8200 Superprobe electron microprobe was used to collect electron backscatter images and EDS spectra of the thin electrode surfaces. The electron beam current was 2*.*5nA, and the hematite samples were sufficiently conductive that no other treatment (i.e. carbon coating) was needed for analysis with the electron microprobe. The composition of the surface was also characterized using a PHI VersaProbe II X-ray photoelectron spectrometer with a scanning monochromated Al source (1486.6 eV; 50 W). The takeoff angle between the sample surface and analyzer was 45°. All data were background subtracted, smoothed using a five-point quadratic Savitzky–Golay algorithm. The binding energy scale was calibrated with respect to the C1s (set to 285 eV). Curve fitting of the Cr 2p_3/2_ peak was performed using Gaussian line shaped on a Shirley-type background. Peak fitting of Cr 2p_3/2_ envelope was performed using previously reported peak parameters^50^. For Cr(III) in Cr_2_O_3_ a single peak definition of the Cr(III) peak was used. Elemental peaks were also fitted to determine shifts in the relative molar abundance of different elements, using the Multipak software provided with the spectrometer.

## Supplementary Information

Supplementary Information 1.

## Data Availability

The datasets generated during and/or analyzed during the current study are available from the corresponding author on reasonable request.
