# Supplementary material for: Semiconducting hematite facilitates microbial and abiotic reduction of chromium
Source: Sci Rep. 2022 May 31;12:9032. doi: 10.1038/s41598-022-12824-y (PMC9156696; doi:10.1038/s41598-022-12824-y)
Supplement: Supplementary file 1 — Supplementary Information 1. [file 41598_2022_12824_MOESM1_ESM.pdf]

Supplementary information for: **Semiconducting hematite facilitates microbial and abiotic reduction of chromium**

Chen, Michael A., Mehta, N., Kocar, Benjamin D.

Additional Materials and Methods

Electrode fabrication

Bulk electrodes consisting of a piece of hematite were made in house by first attaching a gold wire (0.004", 99.99% purity) wound around a supporting plastic ribbon with a conductive silver epoxy (MG Chemicals), with a tightly coiled region that would serve as the connection for an alligator clip. Once the silver epoxy had cured, heat shrink tubing was added to the supporting ribbon and non-conductive epoxy (Devcon 14310) were added to insulate all parts of the electrode except for a single face (opposite the silver epoxy) and a connection point that would remain outside of the experimental chamber. The remaining exposed surface was polished to a flat surface by wet sanding with increasing grits of sandpaper, from 120 grit to 2000 grit. An example electrode is shown in figure 1 in the main text. Hematite sizes varied, but had roughly 1 cm x 2 cm in surface area exposed to solution, and around 1 cm separation between solution surface and the silver epoxy. Electrical attachments to the electrode were made by clamping an alligator clip to the coil of gold wire at the top of the supporting ribbon. A thin section of hematite was also used as an electrode, where a polished thin (1 mm) section of the specular hematite was mounted onto a glass slide with silver epoxy, prepared by Spectrum Petrographics. All edges of the electrode had a bead of conductive silver epoxy that could be used as an electrical connection. Three of these edges were sealed with non-conductive epoxy, the remaining edge was used as the main attachment point by an alligator clip.

Carbon electrodes were made from 1cm thick graphite plates (McMasterCarr), which were cut into 1 cm x 4 cm pieces. Gold wire and a supporting ribbon were attached in a similar fashion as for the bulk hematite electrodes, though only the silver epoxy patch was sealed with non-conductive epoxy. A bulk electrode was used as opposed to a single wire of a conductive material to provide ample surface area for reaction.

Ag/AgCl reference electrodes were fabricated in house for use in potentiostatic experiments, using 99.99% purity silver wire. This wire was coated with AgCl by immersion in a saturated KCl, and exposure to 15V potential with a carbon counter electrode for 10 min. The barrel of the electrode was a glass pipette, where the tip was broken off cleanly and sealed with a small piece of molecular sieve (Malinckrodt, 3Å) held in place with a small amount of super glue The filling solution for these reference electrodes was saturated KCl, and solutions were kept in a saturated KCl solution until use. Reference electrodes were checked against a master Ag/AgCl reference electrode (Microelectrodes, Inc.), and were within 5 mV of the master electrode at all times.

Experimental chambers were custom printed using a Form 2 printer and Clear resin (Form Labs). After removal of support structures needed for printing and sanding of the support attachment points, chambers were coated with a non-reactive two part epoxy and cured following manufacturer instructions (EpoTek 301-2FL). Chambers featured a main filling port with a screw cap, and two holes, all of which could accommodate a 20 mm wide butyl stopper. These stoppers were modified to hold electrodes as required. The chamber was printed in two halves, which sandwiched a rubber gasket and cation exchange membrane (Membranes International, Inc.). The cation exchange membrane was a polymer coated fabric which featured a sulfonic acid functional group, which only allowed for migration of cations to balance charges transferred between the

hematite and carbon electrodes. Membranes were equilibrated with the relevant experimental solution for at least 1 hour before use, and fresh membranes were used in each experiment. Chamber halves were bolted together, and prefilled prior to any experiment to test for a) leakage of solution across the membrane and b) leakage of solution out of the experimental chamber. Only assembled chambers which passed both tests were used. Note that excessive clamping force resulted in chamber breakage.

Cleaning protocols for electrodes and chambers

All electrodes were cleaned prior to use in experiments, first by scrubbing with detergent (Alconox), followed by brief rinsing in 2% HNO_3, a minimum of three rinses with 18MΩ deionized water, then ultrasonication in DI, and finally rinsing with acetone and then isopropanol. Hematite electrodes were re-polished using fresh 2000 grit sandpaper prior to ultrasonication. Carbon and hematite electrodes were then stored dry in clean aluminum foil until use in experiments. Experimental chambers were cleaned by washing with detergent, rinsing in 2% HNO_3, and rinsing with at least 3 times the chamber volume of DI.

Sample measurement

Fe and Cr concentrations were measured in aqueous samples. Aqueous Cr concentrations in samples were measured by ICP-MS using a Sc or Ge internal standard, and samples were diluted by 10 times with 2% HNO_3 to reduce wear on the detector. The choice of internal standard depended on which was most stable over a given run. Triplicate samples were run on the ICP-MS, and sample concentrations did not vary more than 10%. A calibration curve was prepared by dilutions of a multi-element standard (Perkin-Elmer), and ranged from 0.02 μM to 2.0 μM Cr. Fe(II) and total Fe were measured by applying the ferrozine method to the diluted samples with concentration ranges from 18 μM to 180 μM Fe.[1,2] The total solution in experimental chambers

was tracked by measurements in changes of total chamber mass, which was then used to calculate amounts of Fe and Cr in the experimental chambers based on concentration and chamber volume.

Additional results and discussion

Measurements of Hematite Resistivity

Hematite resistivity was measured by coating two sides of a piece of specular hematite with silver epoxy and gold wire, and then measuring the resistance of the piece using a multimeter. Hematite samples were of a similar size and shape as the hematite pieces used as electrodes, but were not the samples used to fabricate the electrodes. Further measurements of hematite resistivity as a function of applied voltage were also taken. This method was applied because hematite is a well-established n-type semi-conductor, which will have shifts in resistivity depending on the applied voltage. Increasing applied potentials should therefore result in band bending that enhances conduction of electrons.[3,4]

The results of these measurements are given in figure S1 for three hematite samples that were of similar size and shape to those used as electrodes. The sample resistance was calculated by first calculating the circuit current and hematite potential drop by the voltage drop over a measurement resistor and thereby the hematite sample resistance. This, with the sample dimensions were then used to estimate the hematite resistivity. The dimensions used to calculate the resistivity assume an idealized rectangular prism geometry, which was reasonably accurate for the samples used. Sample areas did not vary more than 10%, though the sample thickness did vary up to 50% in one sample. Resistivity of the hematite varied both by the voltage applied to the measurement circuit, as well as according to the sample measured. In one sample, the resistivity changed by nearly an order of magnitude in response to an increasing applied voltage,

while for another, little change in resistivity was observed. Hematite orientation in the circuit (both the nearest terminal of the voltage source, and the sample orientation in the circuit) were changed and while some small differences were observed, did not appear to impact the resistivity measured. The measured resistivity by a 2-point resistance measurement across the samples using a multimeter was also a similar value to those produced by resistivity measurements with around 0.5V of applied voltage.

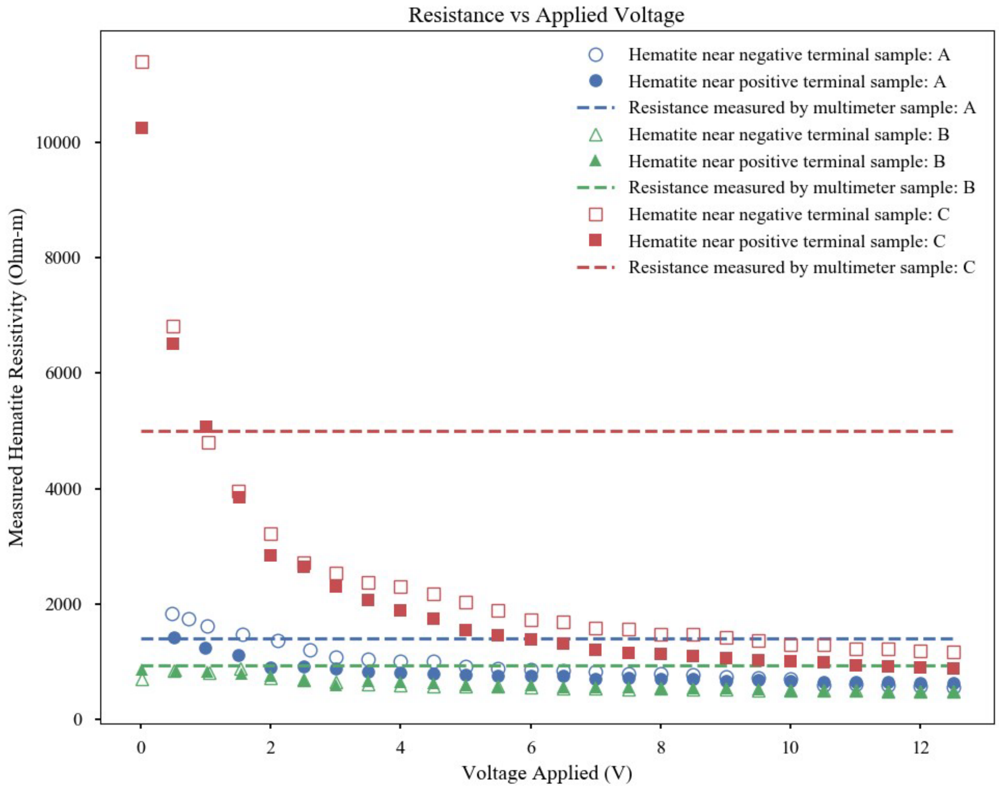

Figure S1: Resistivity measurements of three different samples of specular hematite. Significant variations occur, though the orientation of the hematite with respect to the voltage had less impact compared to the applied voltage and which sample was used. The observed behavior is indicative that the samples were semi conductive, which is expected with hematite.

The measurements of resistivity highlight the large amount of heterogeneity intrinsic to the specular hematite, which is linked to observed variations in the amount of electrons transferred in each experiment. The measured resistivity of specular hematite samples ranged from 500Ωm to nearly 11000Ωm, which falls within the large range of resistivities reported for natural hematite, which cover from 1×10^2 Ωm to 1×10^7 Ωm for bulk hematite.[5] Another electrochemical study that used natural hematite as an electrode, however, reported resistivities of only 0.5Ωm to 10Ωm.[4,6] Surface analysis also helps explain these differences. The electron backscatter images and EDS spectra (figure 3 of main text for backscatter images, and figure S2 here for EDS and backscatter images) of hematite used here clearly show the presence of multiple solids, that would also have markedly different resistivities.

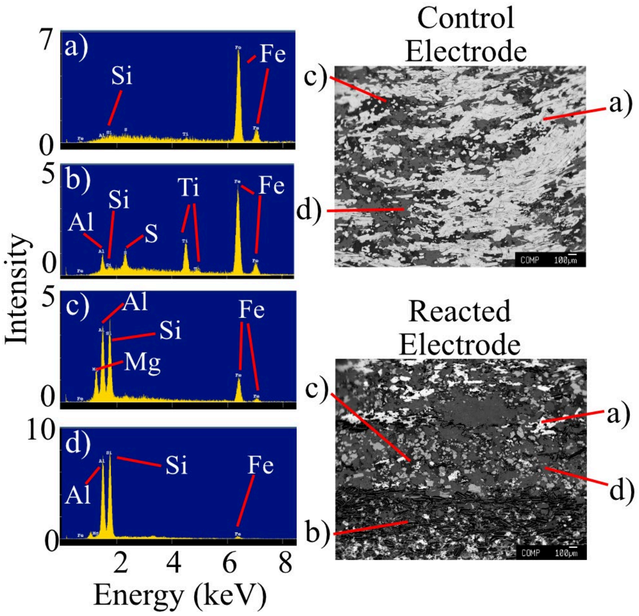

Figure S2 – EDS and electron backscatter images of two electrodes: one which was freshly polished, and one which had been used in an abiotic experiment a potentiostat. EDS spectra corresponding to the four minerals observed in backscatter images can be seen on the left, and the

example of those minerals pointed out on the right. Notable, is that mineral in b) is not found on the unreacted electrode.

Additional surface results

XPS elemental abundance measurements also showed Fe/Si ratios of 0.8 ± 0.7 and Fe/Al ratios of 1.2 ± 1.1 in unreacted samples, further reinforcing that a mixture of solid phases exists in the samples (table 1.2). The heterogeneous distribution of these solids would readily explain the observed variations in hematite sample resistivity. In contrast, neither Si nor Al were reported in the hematite used in the previous electrochemical study, suggesting that the hematite used, while containing some minor impurities, would not have the large variations in resistivity observed here. These differences are also reflected in the reported electrode currents: here, experimental currents were only 2 μA for the biotic experiment, and from 50 μA to μ500 μA in potentiostatic experiments, while maximum currents of 10 μA were observed in the previous cyclic voltammetry studies. It is not immediately apparent why observed currents observed here were larger, when the samples used in that work had significantly less resistivity measured, though this may be a result of differences in the experimental conditions.

The dependence of hematite resistivity on applied voltage may also impact the amounts of current observed in the experiments here. As table 1.1 summarizes, potentiostatic experiments, which poised the hematite electrodes at −1000mV vs. Ag/AgCl, had significantly larger currents compared to biotic experiments. Thermodynamic calculations of *S. Putrefaciens* respiration of lactate give reduction potentials as low as −600mV vs Ag/AgCl.[7] The difference in the reduction potentials shows that *S. Putrefaciens* used in these experiments would likely apply a lower voltage to the hematite electrode, which would immediately result in a lower current, simply by virtue of an increased hematite resistance and the decreased applied voltage. Figure S1 shows that

depending on the hematite sample measured, that difference could also result in an increase of hematite resistance by a factor 2, which would then further decrease current that bacteria could generate. This resistivity increase matches with what would be expected for a semiconducting material, as smaller applied negative potentials result in less charge accumulation in the conduction layer of the semiconductor that in turns allows for less current.[3,4]

Cr control experiments

Experiments were performed to analyze potential sources of Cr removal from solution when no current was being provided to the hematite electrode. Two processes were considered: sorption of Cr to chamber surfaces in the absence of an electrical connection between a hematite and carbon electrode, and loss of Cr resulting from any background current that might develop. Sorption losses were tested in an experimental chamber set up for a biotic experiment, but no electrical connection between the carbon and hematite electrodes was made. Thus, any changes in Cr concentration had to result from sorption to experimental surfaces. To test for the presence of background current, a biotic experiment was performed with an electrical connection between the carbon and hematite electrodes, but the spike of metal reducing bacteria was omitted. This experiment was performed using a chamber that had been used for a sorption experiment previously to reduce any possible sorption that might affect the results. The results of all 4 experiments are plotted in figure S3.

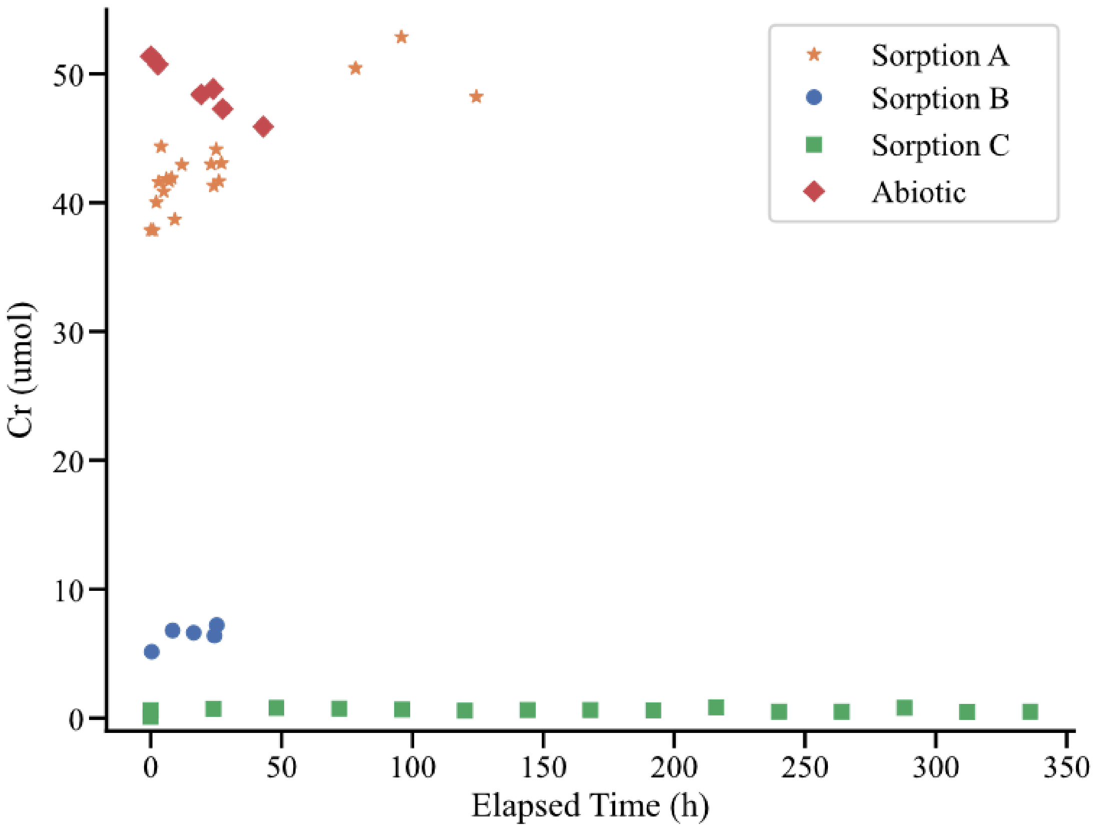

Figure S3 - Control experiment results. Data sets marked “sorption” were performed in an experimental chamber without an electron source and no connection between the carbon and hematite electrodes. In contrast, the abiotic experiment made the connection between the electrodes, but omitted an electron source.

Three experiments to test sorption were performed: The first was performed at a significantly higher Cr concentration (~45 umol total Cr at the start, labeled Sorption A in figure S3) and 125 hours. This represented a time period close to that used in potentiostatic experiments, and at a very high concentration as it would readily show sorption resulting from mass action. A second was performed for 24 hours and at about 7 umol total Cr at the start (Sorption B in figure

S3). This experiment closely matched the initial concentrations used in experiments. The last experiment was performed at a lower concentration for 300 hours, matching the maximum experimental length (Sorption C in figure S3). These three experiments bracket the range of Cr concentrations and time periods that were used. In sorption experiments, no decreases in Cr are observed. The highest concentration experiment, curiously, had an increase of Cr over time, which is most likely due to incomplete mixing of the initial Cr spike (the final Cr concentration matches what was expected based on the spike concentration). In the experiment with electrical connection (labeled Abiotic in figure S3), no current is observed between the carbon and hematite electrodes, and Cr concentrations appear to decrease up to 5 μmol.

The results of the control experiments are used to highlight any chemical or physical reactions that may be impacting removal of Cr observed during potentiostatic and biotic experiments. Sorption control experiments clearly show no decrease in Cr concentration, thus sorption does not affect the observed removal of Cr in experiments in the main text. Even if sorption was occurring to the reacted electrode, Cr species would likely be reduced by either Fe(II) generated from the electrode, or directly by donated electrons. The lack of observed sorption matches with previous studies of Cr sorption to Fe (hydr)oxide solids in general, where only $\mu mol/m^2$ amounts of Cr sorption were observed as well.[8,9] The mechanical polishing of the electrodes, as well as the relatively small contact area, in combination with previously reported sorption rates, show that even if sorption is occurring, it would have a minimal impact on the experimental results.

The results of the abiotic control experiment showed up to 5 μmol decrease in Cr from the start to the finish of the experiment. Because this was performed in a chamber that had previously been used in a sorption experiment (specifically, experiment Sorption A), sorption likely does not

play a role in this observed decrease. Similarly, because no current was observed, it is not likely that Cr reduction is occurring as a result of electrons delivered from the carbon electrode. The total loss (5 μmol) is within 10% of the total, which is within the uncertainty of the ICP-MS measurement. Another possibility is that exposure to light is leading to Cr reduction, via a photoreduction mechanism, as hematite has some photoreduction capacity.[10,11] In those cases, an external electron source was needed to drive the first step of reduction (i.e. reduction of Cr(VI) to Cr(V)), and the remaining reduction to Cr(III) proceeds with the assistance of light. Since there

was no current source recorded in this work, this mechanism is likely not driving Cr reduction. Similarly, photoreduction is unlikely due to the relatively low purity and thickness of the hematite electrodes used. Since this was not controlled for explicitly, however, the possibility remains. While it is not clear the exact source of the decrease, the amount is small enough (10% of initial) that even if this separate reduction mechanism was operating in this experimental system, the indirect reduction by microbes or the potentiostat would still occur.

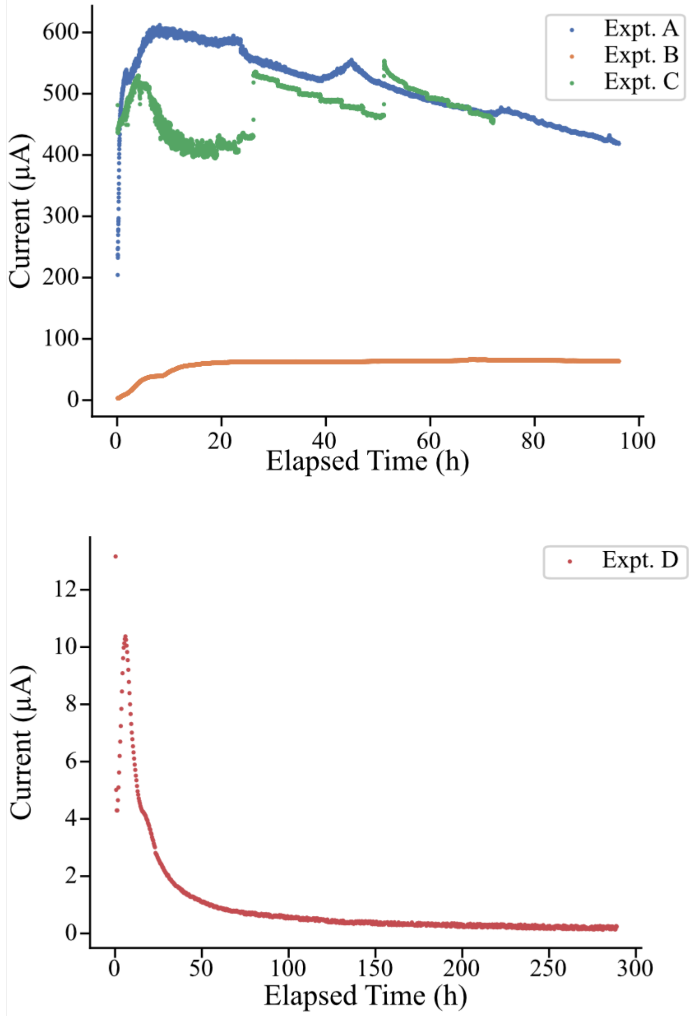

Figure S4 – Current measured in potentiostatic (top) and biotic (bottom) experiments. The integrated current is used to calculate the total transfer of electrons

Current measurements

Plots of the measured current are given in figure S4 for experiments A-D. Potentiostatic experiments A and C, which showed significantly larger transferred electrons compared to experiment B, show an initial spike in current, followed by a decrease in current, with jumps every 30 hours or so. Experiment B does not show these variations, but rather a steady current after the initial increase. For biotic experiments, D, a rapid current increase occurs near the beginning,

and then it decreases to a relatively stable amount for the remainder of the experiment, around 2-3 µA for both experiments.

The source of initial spikes in current is most likely associated with the initial equilibration of the experimental system to the voltage applied by either the potentiostat and the microbes, however, it becomes less clear what causes the subsequent increases in current observed in the potentiostatic experiments. One possibility is that changes in the mineral surface due to the high rate of Fe reduction is leading to changes in resistance at the surface. The natural variabilities in the hematite, as well as the electron imaging support this possibility. However, voltametric experiments using these particular hematite samples may further elucidate the source of these spikes in current, however, they do not influence the overall conclusions linking hematite, Cr, and Fe discussed in the main text.

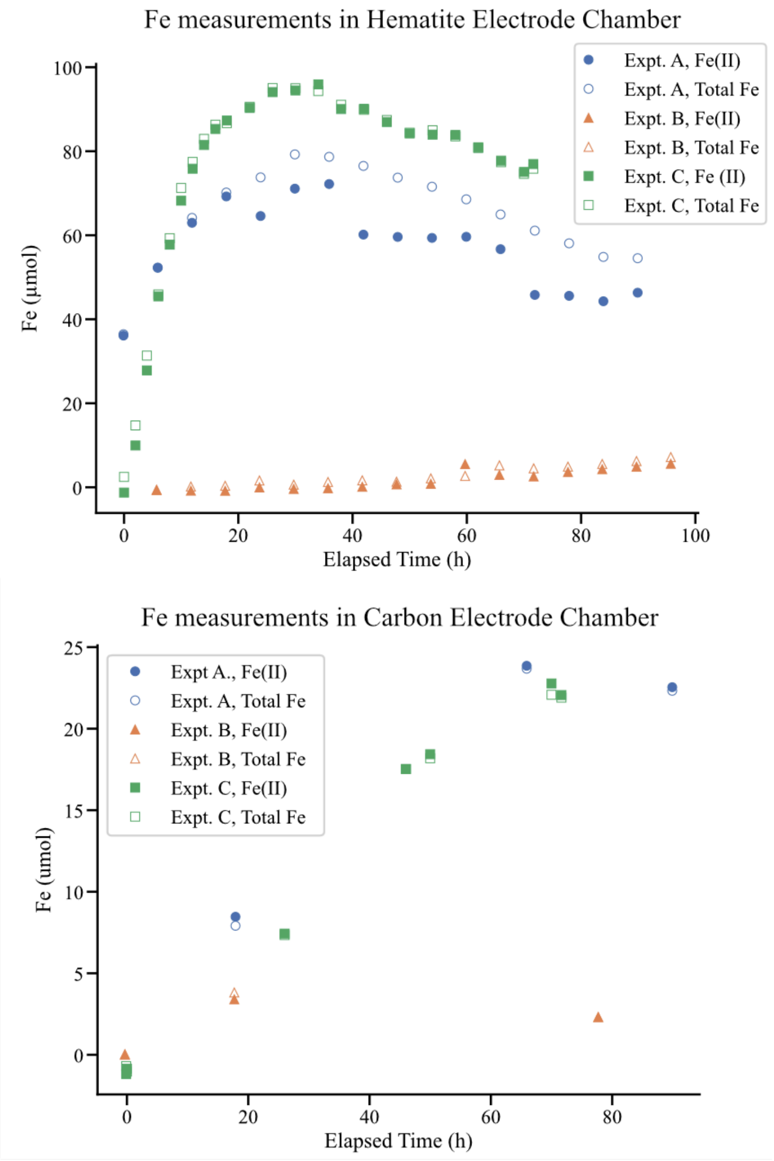

Figure S5 – Fe measurements from potentiostatic experiments. Top image gives Fe measurements for the chamber containing the hematite electrode, while the bottom image gives measurements of Fe from the carbon electrode

Fe measurements and Fe movement in experimental systems

Fe(II) was also observed in the chamber containing the carbon electrode in these experiments at lower concentrations than observed in the chamber holding the hematite electrode (figure S5). This indicates that Fe(II) had crossed the cation exchange membrane, however, this did not appear to interfere with electron transfer through the carbon electrode, which still resulted in Fe reduction in the hematite chamber (figure S4). The amount of Fe that has reached the other chamber does not complete the total balance of Fe that would be expected based on the delivered current to the hematite electrode, thus the most likely remaining sink for Fe(II) produced in these experiments must be association with the hematite surface. Further study, particularly with labeled isotopic tracers, would help complete the balance of Fe observed in these systems, but the overall phenomena do not strongly affect the observed Cr reduction nor the mechanism proposed in the main text which links Fe and Cr.

XPS Results

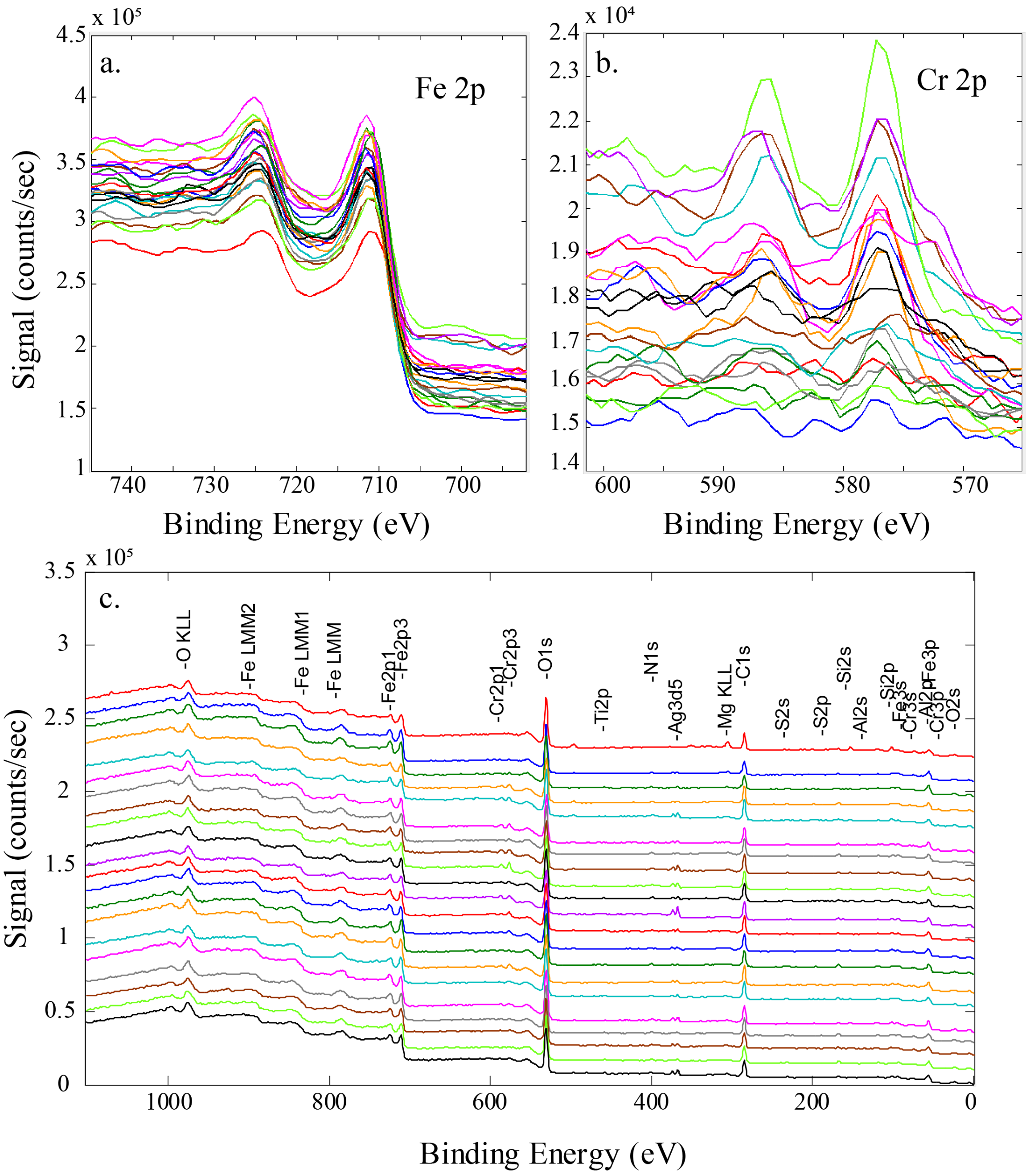

Figure S6: XPS spectra collected from various points on the reacted electrode. Spectra of the Fe 2p and Cr 2p (a. and b.) are shown to highlight variations in the quantification of Fe and Cr. Spectra are presented after background subtraction. Peaks for identified elements are identified in the spectra in c., where the full range of collected energies are presented. Unreacted electrodes displayed no measurable Cr 2p peak.

Results and spectra collected from XPS measurements of an unreacted thin hematite electrode and reacted hematite electrode are presented in figure S5. The epoxies used in electrode construction degassed readily and inhibited XPS analysis as the degassing epoxy posed a risk to the ultra-high vacuum sensitive equipment. This was overcome in the thin electrode, where minimal epoxy was used. The points that spectra were taken at are presented in figure S6. Background subtraction and peak fitting for elements present using the Multipak software provided elemental abundance results, which are given in table S1 and S2 for both an unreacted and reacted electrode, respectively. While this does not provide quantitative measurements of total amounts of a given element, elemental ratios can be used to discern shifts in the mineralogy of the sample. In the unreacted sample, no Cr was present, however, in the reacted sample, Cr is detected, with Cr/Fe ratios that varied from 0 (no Cr) to 0.5. An increase in the Fe/Si is also observed from unreacted to the reacted electrode, while minimal change in Fe/O ratios is observed. The fraction of Cr present was variable throughout the single unreacted electrode, evidenced by Cr 2p spectra, where none of the characteristic peaks are observed, while in some, the peaks are clearly seen. This, in parallel with the observed mineralogical variation observed in the electron microprobe images, strongly suggests that there is a heterogenous surface reactivity, in which certain locations are preferentially releasing Fe(II) compared to others.

Examination of the Fe 2p 3/2 peak (Figure S8) showed no meaningful shifts when comparing the pre and post reaction electrodes, with both peaks primarily corresponding to Fe(III). While this appears contradictory to the geochemical results which clearly show the evolution of Fe(II) by reduction, there are a few reasons that a corresponding shift might not be observed. First, the peak locations that distinguish various Fe(II) and Fe(III) solids are very close, with even different Fe(III) solids having very close peaks to Fe(II) solids.[12] The collected XPS data and

analysis lack the sensitivity to discriminate between these many solids. Second, while there was evidence of some kind of surficial mineral transformation, there was insufficient current delivered to completely remove all of the original hematite surface. The remaining hematite then may be dominating the XPS signal observed, despite the release of Fe(II), which would readily result in no changes in the XPS peak. Therefore, no peak fitting was performed on the Fe XPS spectra.

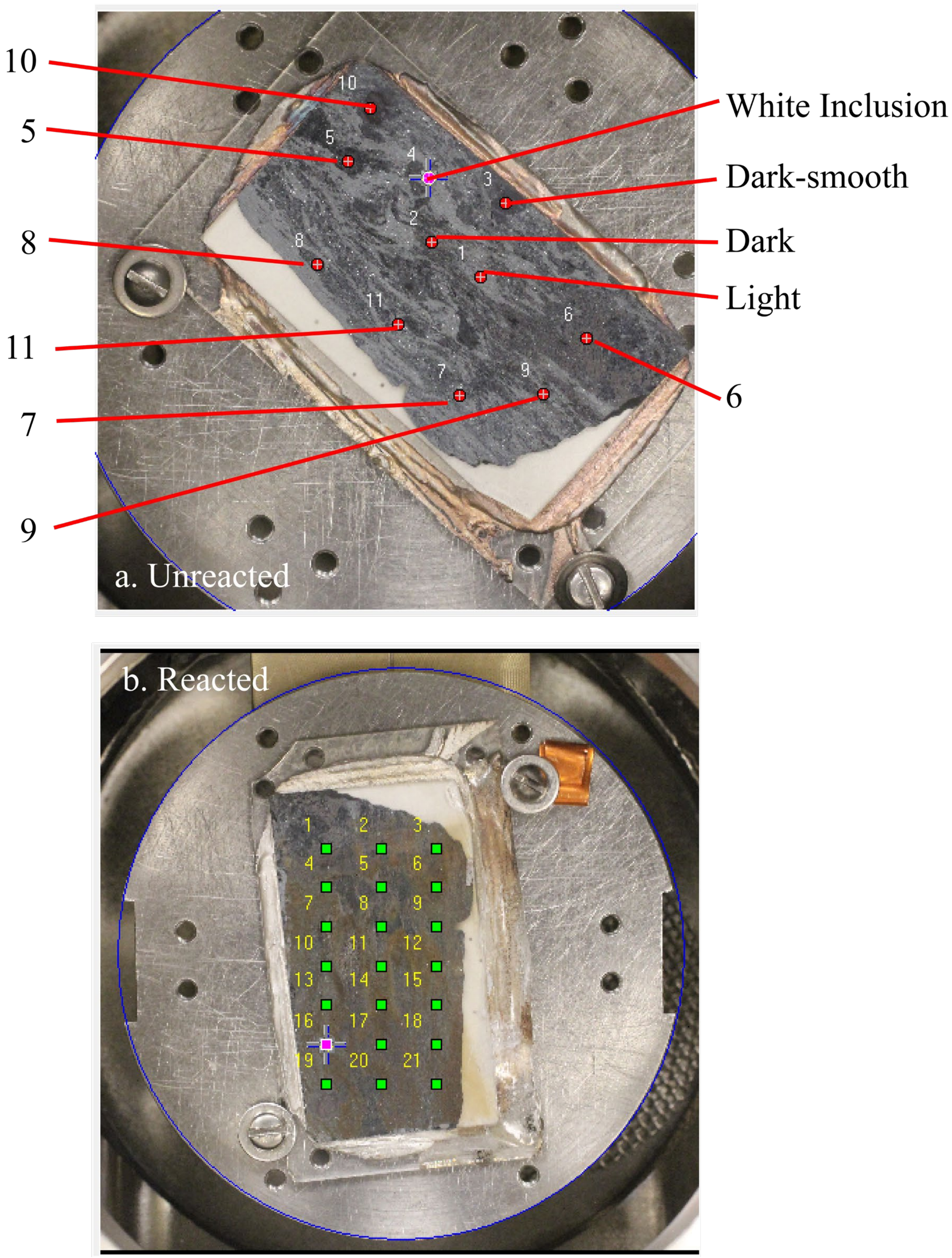

Figure S7: Locations of XPS spectra for both the reacted and unreacted electrodes.

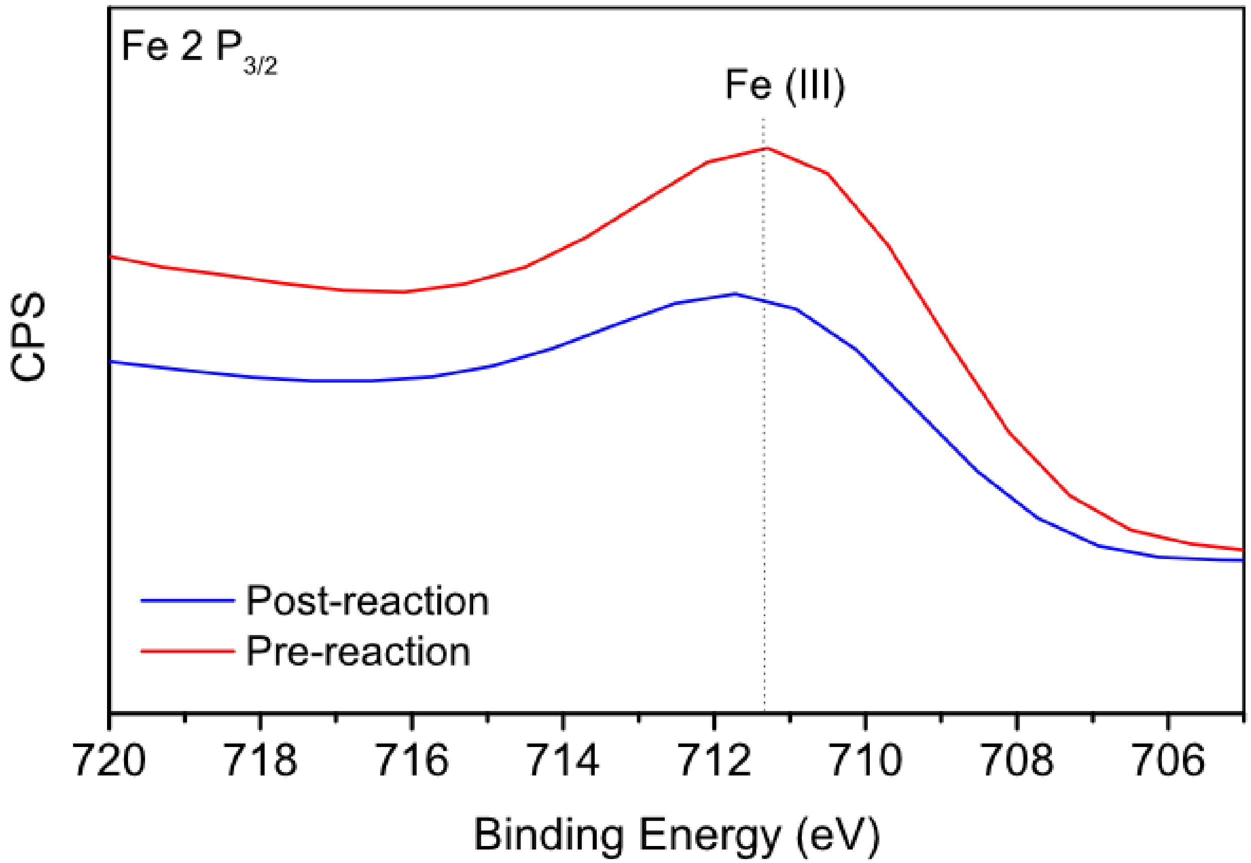

Figure S8 – Fe 2p 3/2 spectra, which show a predominantly Fe(III) peak, and no evidence of an Fe(II) peak.

Point	C	N	O	Na	Mg	Al	Si	K	Ca	Mn	Fe
Light	32.31	2.41	47.83	5.66		2.23	3.50			0.24	5.82
Dark	29.61	1.55	52.61	2.13		3.41	6.24			1.62	2.82
Dark-smooth	15.72	1.48	55.94	4.15	1.20	8.13	10.46	1.89	0.47		0.55
Point 5	13.73	1.55	58.59	2.12	3.00	5.08	12.57	0.83	0.53		1.99
Point 6	17.53	2.28	56.97	3.60		5.73	7.36	0.84	1.05		4.64
Point 7	17.52	1.82	58.08	1.62		7.53	8.95	0.83	0.66		2.99
Point 8	17.09	1.70	59.53	3.77		4.65	6.28	1.13	0.78		5.07
Point 9	20.91	3.07	55.64	5.75		2.42	4.02	0.87	0.21		7.11

Point 10	17.46	2.12	57.51	2.97		9.18	7.38	0.72	0.33	0.23	2.09
Point 11	26.82	3.06	51.60	3.54		2.80	3.92	0.20	0.26	0.29	7.51
White inclusion	48.98	2.30	37.02	1.84		2.30	4.38				3.18
Mean	30.96	1.98	50.22	3.90	2.82	2.8	4.9	0.9	0.5	0.9	4.3
Standard deviation	1.9	0.6	3.4	2.5	0.9	0.8	1.9	0.4	0.3	1.0	2.1

Table S1: Elemental abundances on the unreacted thin electrode determined by XPS. Abundances are relative and determined by fitting peaks in observed spectra. Blank values mean that no peak was fitted, and the element was not detected.

Point	C	N	O	Si	K	Ca	Cr	Fe	Cr/Fe
1	33.8	0.3	49.6	4.3	2.6	0.8	0.9	7.6	0.1
2	34.1	1.7	51.4	0.8	0.7	0.2	0.3	10.9	0.0
3	33.0	1.5	53.4	0.9	0.3		0.3	10.6	0.0
4	41.4	1.4	46.9	1.4	0.3	0.1	2.6	6.0	0.4
5	41.6	2.0	45.5	2.9	0.8	0.1	2.2	5.1	0.4
6	38.1	2.0	48.5	1.3	0.3		2.4	7.5	0.3
7	38.9	2.0	47.8	1.1	0.6		1.1	8.5	0.1
8	41.1	2.4	46.2	1.8	0.5		2.8	5.2	0.5
9	39.5	1.4	48.5	0.8	0.1	0.1	3.2	6.3	0.5
10	41.1	1.9	47.6	1.7	0.6		0.8	6.3	0.1
11	42.8	1.6	45.0	1.2	0.4	0.2	2.4	6.5	0.4
12	40.7	1.8	47.5	1.5	0.4		1.7	6.5	0.3

13	39.2	0.8	47.5	2.3	0.6		1.9	7.8	0.2
14	36.2	2.1	49.7	0.7	0.3		0.8	10.2	0.1
15	41.5	1.1	46.6	1.5	0.6		1.6	7.3	0.2
16	39.4	2.1	48.1	0.6	0.6	0.1	0.6	8.5	0.1
17	39.0	2.1	47.4	1.6	0.6	0.2	1.1	8.0	0.1
18	39.1	1.7	48.8	0.9	0.4		0.5	8.6	0.1
19	37.9	2.0	49.2	2.2	0.7	0.1	0.7	7.4	0.1
20	40.3	2.2	47.1	0.8	0.3	0.3		9.0	0.0
21	39.8	1.0	48.1	1.7	0.6		0.9	7.8	0.1
Mean	39.0	1.7	48.1	1.5	0.6	0.1	1.4	7.7	
Standard Deviation	2.7	0.5	1.9	0.9	0.5	0.2	0.9	1.6	

Table S2: Elemental abundances on the reacted thin electrode determined by XPS. As with the unreacted electrode, blank values indicate no peak was fitted. Cr abundances varied, as can be seen the variation of peak strength in figure S5, but was clearly observed at some locations on the electrode.

References

1. Stookey, L. L. Ferrozine---a new spectrophotometric reagent for iron. *Analytical Chemistry* **42**, 779–781 (1970).
2. Viollier, E., Inglett, P. W., Hunter, K., Roychoudhury, A. N. & Van Cappellen, P. The ferrozine method revisited: Fe(II)/Fe(III) determination in natural waters. *Applied Geochemistry* **15**, 785–790 (2000).

3. Xu, Y. & Schoonen, M. A. A. The absolute energy positions of conduction and valence bands of selected semiconducting minerals. *American Mineralogist* **85**, 543–556 (2000).
4. Meitl, L. A. *et al.* Electrochemical interaction of Shewanella oneidensis MR-1 and its outer membrane cytochromes OmcA and MtrC with hematite electrodes. *Geochimica et Cosmochimica Acta* **73**, 5292–5307 (2009).
5. Yanina, S. V. & Rosso, K. M. Linked Reactivity at Mineral-Water Interfaces Through Bulk Crystal Conduction. *Science* **320**, 218–222 (2008).
6. Eggleston, C. M. *et al.* The structure of hematite (α-Fe2O3) (001) surfaces in aqueous media: scanning tunneling microscopy and resonant tunneling calculations of coexisting O and Fe terminations. *Geochimica et Cosmochimica Acta* **67**, 985–1000 (2003).
7. Bianco, P. & Haladjian, J. Recent progress in the electrochemistry of c-type cytochromes. *Biochimie* **76**, 605–613 (1994).
8. Johnston, C. P. & Chrysochoou, M. Mechanisms of chromate adsorption on hematite. *Geochimica et Cosmochimica Acta* **138**, 146–157 (2014).
9. Deng, Y., Stjernström, M. & Banwart, S. Accumulation and remobilization of aqueous chromium(VI) at iron oxide surfaces: Application of a thin-film continuous flow-through reactor. *Journal of Contaminant Hydrology* **21**, 141–151 (1996).
10. Whitaker, A. H., Peña, J., Amor, M. & Duckworth, O. W. Cr(VI) uptake and reduction by biogenic iron (oxyhydr)oxides. *Environ. Sci.: Processes Impacts* **20**, 1056–1068 (2018).
11. Tamirat, A. G., Rick, J., Dubale, A. A., Su, W.-N. & Hwang, B.-J. Using hematite for photoelectrochemical water splitting: a review of current progress and challenges. *Nanoscale Horiz.* **1**, 243–267 (2016).

12. Biesinger, M. C. *et al.* Resolving surface chemical states in XPS analysis of first row transition metals, oxides and hydroxides: Cr, Mn, Fe, Co and Ni. *Applied Surface Science* **257**, 2717–2730 (2011).
